# PET imaging of TREM2 in amyloid-beta induced neuroinflammation

**DOI:** 10.1007/s00259-025-07358-0

**Published:** 2025-05-28

**Authors:** Amelia D Dahlén, Sahar Roshanbin, Ximena Aguilar, Nadja M Bucher, Sara Lopes van den Broek, Dag Sehlin, Stina Syvänen

**Affiliations:** Department of Public Health and Caring Sciences, Section of Molecular Geriatrics, Uppsala University, Uppsala, Sweden

**Keywords:** Neuroinflammation, Antibody-based PET, Alzheimer’s disease, Bispecific antibody, The blood-brain barrier

## Abstract

**Purpose:**

The triggering receptor expressed on myeloid cells 2 (TREM2) has become a promising target for biologics in both monitoring and treating neuroinflammation in Alzheimer’s disease (AD). This study aimed to develop and compare bispecific anti-TREM2 antibodies featuring different transferrin receptor (TfR) binders to enhance brain delivery, identifying the most suitable format for in vivo PET imaging of TREM2 in transgenic AD mice.

**Methods:**

Three bispecific TREM2-antibody formats were produced and evaluated for their ability to cross the blood-brain barrier (BBB) via TfR-mediated transcytosis and bind TREM2. Blood concentration profiles up to 72 h post-injection (p.i.), and ex vivo brain uptake of iodine-125-labeled antibody constructs were quantified in App^NL−G−F^ and age-matched wild type (WT) mice using a γ-counter. The best-performing bispecific TREM2-antibody was radiolabeled with iodine-124 and used for in vivo PET imaging of brain TREM2 levels in App^NL−G−F^ mice at 72 h p.i. Brain TREM2 concentrations were subsequently quantified using ELISA.

**Results:**

The antibody format carrying two scFv8D3 TfR-binders (IgG-scFv_2_), demonstrated the highest brain concentrations of all tested bispecific constructs. This antibody also exhibited significantly higher brain concentrations in App^NL−G−F^ mice compared to WT mice at both 48 and 72 h p.i. This difference was further visualized and quantified through in vivo PET imaging. Moreover, brain concentrations of the antibody ligand correlated with elevated TREM2 levels in brain homogenates.

**Conclusion:**

These findings highlight IgG-scFv_2_ as a promising radioligand for in vivo PET imaging of TREM2, advancing non-invasive neuroinflammation studies and supporting drug development for AD and other neurodegenerative diseases.

**Supplementary Information:**

The online version contains supplementary material available at 10.1007/s00259-025-07358-0.

## Introduction

In the short time span of twelve months, two antibody-based treatments for Alzheimer’s disease (AD) have been approved by the Food and Drug Administration (FDA). Lecanemab by Eisai/BioArctic and Donanemab by Eli Lilly have both been successful in slowing cognitive decline and the removal of neuropathological amyloid-beta (Aβ) protein in the AD brain [1, 2]. These results build on the progress of continually evolving AD biomarkers, with positron emission tomography (PET) imaging being particularly notable. The use of amyloid radioligands to monitor the reduction of brain Aβ during immunotherapy has advanced AD research, opening up for new treatment possibilities that go beyond purely symptomatic interventions.

With this said, AD is a multifaceted disease likely requiring multiple lines of treatment to combat the neurodegenerative processes. Alongside the toxic species of Aβ and neurofibrillary tau tangles, neuroinflammation is now a widely recognized component of AD [3]. The accumulation of Aβ may initiate neuroinflammatory cascades of cytokines and chemokines, mediated by microglia and astrocytes of the central nervous system (CNS), which contribute to the disease severity [4]. Thus, neuroinflammation in AD calls for its own treatment approach. Capturing neuroinflammation through in vivo imaging, e.g. PET radioligands, could therefore carry great diagnostic potential for AD patients and for the development of new anti-inflammatory drugs.

Mitochondrial 18-kDa translocator protein (TSPO) is the most commonly investigated target for PET imaging of neuroinflammation, and has been visualized using [^11^C]PK11195, [^18^F]DPA-714, [^11^C]PBR28 and [^18^F]GE-180 among others [5–8]. However, interpretation of TSPO PET imaging is confounded by large inter-individual variability in binding affinity due to the genetic polymorphism rs6971 [7, 9]. Less polymorphism sensitive radioligands, such as [^18^F]BS224, have been gaining traction as third generation TSPO-tracers [10]. Nevertheless, the interpretation of TSPO PET is hampered by TSPO being expressed in both microglia and astrocytes in AD and non-AD brains [11].

Another neuroinflammation target is monoamine oxidase B (MAO-B), which metabolizes dopamine and histamine in the brain [12]. It is localized in the mitochondrial membrane of astrocytes and is targeted by radioligands such as [^11^C]DED [13]. Upon its covalent binding to MAO-B, [^11^C]DED irreversibly inhibits MAO-B’s enzymatic actions and is therefore referred to as a suicide enzyme inactivator [13]. PET-imaging with [^11^C]DED captures elevated MAO-B in early disease stages, but does not reflect astrogliosis as the Aβ burden grows [14, 15]. Instead, the PET-ligand [^11^C]BU99008 that binds to the Imidazoline 2 Binding Site (I2BS) on the astrocytic mitochondrial membrane may be better at capturing the later stages of astrogliosis [16]. For example, [^3^H]BU99008 shows higher post mortem binding in brain sections from sporadic AD cases in comparison to control cases [17]. Still, the heterogeneity of both microglia and astrocytes throughout the disease progression makes it difficult to follow the neuroinflammatory trajectory. Thus, there is an unmet need for new improved and specific PET radioligands for the visualization of neuroinflammation.

Triggering receptor expressed on myeloid cells 2 (TREM2) is expressed by microglia and regulates the inflammatory response by dampening the secretion of pro-inflammatory cytokines and improving microglial phagocytosis of Aβ [18, 19]. Several point mutations in the *TREM2* gene have been linked to AD: the most notable amino acid substitution, R47H, leads to a four-fold increased risk of developing late-onset AD [20, 21]. Sporadic AD patients carrying TREM2 risk variants have a higher percentage of dystrophic microglia, a phenomenon recapitulated in preclinical studies where TREM2 deficiency leads to microglial accumulation of ApoE and impaired microgliosis [22, 23]. Upon binding of TREM2’s ligands, such as Aβ and apolipoprotein E (ApoE), to the extracellular domain, the intracellular adapter protein DNAX-activation protein 10 or 12 (DAP10/12) binds to the cytoplasmic tail of TREM2 to initiate downstream signaling [18]. The extracellular domain of TREM2 can initiate parallel inflammatory pathways following cleavage by a disintegrin and metalloproteinase domain-containing protein (ADAM10 and 17) at histidine 157 and serine 158, resulting in soluble TREM2 (sTREM2) [24]. Although the functions of sTREM2 are not fully delineated, it may negatively impact the anti-inflammatory processes prompted by TREM2 activation, as fewer ligands can bind to the ectodomain [25].

With growing interest in developing therapeutics to mitigate neuroinflammation in AD, antibody-based PET radioligands targeting TREM2 present a promising alternative to the aforementioned small-molecule radioligands for visualizing activated microglia. However, to use antibodies as brain PET radioligands, their inherently low brain delivery across the blood-brain barrier (BBB) has to be increased. This can be achieved by engineering antibodies into bispecific formats to also target the transferrin receptor 1 (TfR1, hereafter TfR) expressed at the BBB. Thus, TfR acts as a shuttle for the antibody and can increase antibody brain concentrations by 10- to 80-fold, depending on the dose and the bispecific format used [26–30]. We have previously demonstrated that such bispecific antibodies, after radiolabeling, can serve as PET radioligands to detect proteins like Aβ and alpha-synuclein in the living brain [31–37]. Prior preclinical TREM2 antibody-based neuroimaging studies have used [^124^I]-labeled AF1729 in tg-ArcSwe mice and [^64^Cu]-labeled 4D9 in 5xFAD mice [38, 39]. The antibody-based radioligands described in this study are based on the antibody 14D3 [40], which binds the N-terminal of TREM2 and inhibits the cleavage of sTREM2.

The aim of this study was to explore and compare bispecific formats of the anti-TREM2 antibody, with the ultimate goal of developing a PET ligand for in vivo visualization and quantification of TREM2. This work contributes to advancing PET imaging tools for studying neuroinflammatory markers in neurodegenerative diseases.

## Materials and methods

### Antibody design

**Fig. 1:**
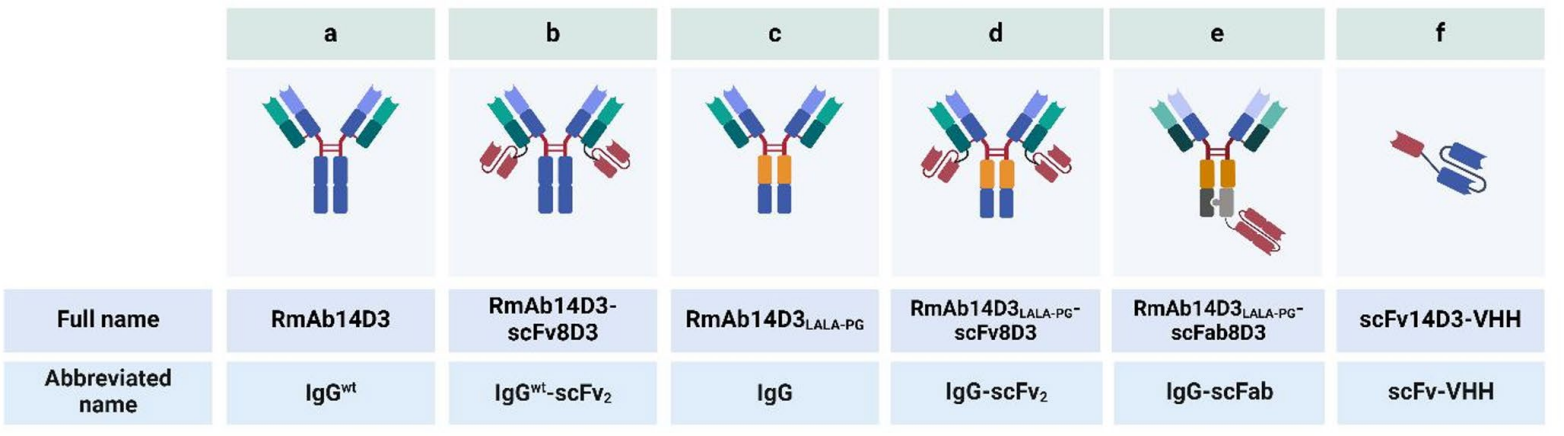
Produced anti-TREM2-anti-TfR constructs

The antibodies were designed based on the amino acid sequence of the variable domains of monoclonal antibody 14D3 (Fig. 1), which binds between amino acids 148–166 of murine membrane bound TREM2 and between amino acids 148–166 of human membrane bound TREM2 [40]. Monospecific TREM2 antibodies, IgG^wt^ (RmAb14D3, 148 kDa) and IgG (RmAb14D3_LALA−PG_, 148 kDa), had murine IgG2c backbones suitable for Bl6 mouse lines (Fig. 1a, c). IgG^wt^-scFv_2_ (RmAb14D3-scFv8D3, 203 kDa) and IgG-scFv_2_ (RmAb14D3_LALA−PG_-scFv8D3, 203 kDa) contained bivalent single-chain variable fragments (scFv) of the TfR binding antibody 8D3 fused to the c-termini of the light chains [27, 41] (Fig. 1b, d). The monovalent knob-in-hole antibody IgG-scFab (RmAb14D3_LALA−PG_-scFab8D3, 206 kDa) contained a single-chain Fab fragment (scFab) of 8D3 attached to the c-terminus of the heavy chain [28, 30] (Fig. 1e). The smallest scFv-VHH (scFv14D3-VHH, 41 kDa), consisted of a TfR Nanobody of camelid origin coupled via a linker to scFv14D3 [42] (Fig. 1f). IgG, IgG-scFv_2_ and IgG-scFab contained the LALA-PG mutations, which eliminate Fcγ receptor binding to reduce antibody-dependent cellular cytotoxicity [43] (Fig. 1c-e). For antibody production and purification protocols, please see Supplementary Information.

### Enzyme-linked immunosorbent assays (ELISA)

To assess the post-production binding affinity of the antibodies to murine TfR protein and murine TREM2 protein, indirect ELISAs were performed in half-area 96-well plates (Corning inc.). The plate was coated with the following proteins diluted in phosphate buffered saline (PBS): murine TfR protein (0.5 µg/mL, in-house produced), murine TREM2 protein (5 µg/mL, SinoBiological, 158–50149-M02H-100) and human TREM2 protein (0.5 µg/mL, SinoBiological, 158–11084-H08H-100). Plates were incubated overnight at 4 °C. Following incubation, the plates were blocked with 1% bovine serum albumin (BSA) for 2 h at RT on a shaker. Biotinylated bispecific antibodies were added in 5x dilution series, staring at 250 nM in ELISA incubation buffer (PBS with 0.1% BSA, 0.05% Tween, and 0.15% Proclin), and incubated overnight at 4 °C. Horseradish peroxidase (HRP) conjugated streptavidin (Mabtech, 3310-9-1000) was then added at a 1:2000 dilution for 1 h at RT, on a shaker.

To compare binding affinity before and after radiolabeling, the plate was coated with the following proteins and antibodies, diluted in PBS: anti-mouse IgG (0.5 µg/mL, VECTOR, AI-2000-1.5), anti-human IgG (0.5 µg/mL, Mabtech, MT91/145), StrepMAB-Classic (0.5 µg/mL, Iba Life Sciences, 2–1507-001), murine TfR protein (0.5 µg/mL, in-house produced), and murine TREM2 protein (5 µg/mL, SinoBiological, 158–50149-M02H-100). Antibodies were added in 5x dilution series, staring at 50 nM in ELISA incubation buffer, and incubated overnight at 4 °C. Detection antibodies, including anti-mouse-F(ab’)_2_-HRP (Jackson ImmunoResearch Laboratories, 115-035-006), anti-human-F(ab’)_2_-HRP (Jackson ImmunoResearch Laboratories, 109-036-006), StrepMAB-Classic HRP (Iba Lifesciences, 2–1509-001) or Anti-Camelid VHH Antibody [HRP] (MonoRab™, A01861) were added at a 1:2000 dilution for 1 h at RT, on a shaker.

K blue aqueous TMB substrate (Neogen Corp., Lexington, USA) was used to develop ELISA signals and the reaction was stopped with 1 M H_2_SO_4_. The plates were read with a spectrophotometer at 450 nm.

### Animals

Knock-in App^NL-G-F^ mice [44], expressing human APP with the Swedish (KM670/671 NL), Arctic (E693G) and Beyreuter/Iberian (I716 F) mutations and wild-type (WT) mice (C57BL/6 JBomTac) were used for in vivo (*n* = 8) and ex vivo (*n* = 99) studies (Supplementary Table S1). Young mice (4–5 months) were used in the initial brain uptake studies and aged mice (11.5–19 months) were used in subsequent brain retention studies. Both male and female animals were included (Supplementary Table S1). App^NL-G-F^ male mice weighed 40.1 ± 4.2 g, App^NL-G-F^ female mice 29.9 ± 4.7 g, WT male mice 36.9 ± 4.8 g and WT female mice 29.7 ± 5.0 g. The animals were housed in an approved animal facility at Uppsala University with *ad libitum* access to food and water. The study adhered to the Animal Research: Reporting of In Vivo Experiments (ARRIVE) guidelines. Two animals were excluded throughout the study; one mouse died during the CT scan and another had an incomplete perfusion. All described procedures were approved by the Uppsala Country Animal Ethics board (5.8.18–20401/2020) following the legislation and regulations of the Swedish Animal Welfare Agency and European Communities Council Directive of 22 September 2010 (2010/63/EU).

### Radiolabeling

The Chloramine-T method was used to radiolabel the antibodies by direct iodination with iodine-125 (^125^I) (half-life 59.5 days) [45]. The antibody was mixed with [^125^I]NaI (Perkin-Elmer Inc Waltham, MA, USA) and 5 µg (200 µM in PBS) Chloramine-T (Sigma Aldrich) in filtered PBS. After 90 s, the reaction was quenched by the addition of 10 µg (440 µM in PBS) sodium metabisulfite (Sigma Aldrich). On average, 95.5 ± 75 µg of antibody was labeled with 14.2 ± 9.5 MBq of ^125^I. The resulting product was separated from free iodine and low molecular weight products using Zeba spin desalting columns (7 K MWCO, 0.5 mL, 89882, Thermo Fisher) or diluted to 500 µl with PBS and passed through a NAP-5 size exclusion column (5 kDa cutoff, GE Healthcare, Uppsala, Sweden) with a total elution volume of 1 mL PBS. The average radiochemical yield was 69.6 ± 10.0%.

For the PET study, 75.4 MBq of [^124^I]NaI (half-life 4.2 days) (Advanced Center Oncology Macerata, Montecosaro, Italy) was preincubated with 100 µM NaI (3.4 µM in final volume), for 15 min. The iodine solution was neutralized with 36 µl 0.5% acetic acid (83.3 mM) and 26 µl 10x PBS, before addition of 420 µg of IgG-scFv_2_ (5.6 µg/MBq) in filtered PBS and 38 µg Chloramine-T (Sigma Aldrich) to a final reaction volume of 517 µl. After 120 s, the reaction was quenched by the addition of 76 µg sodium metabisulfite (Sigma Aldrich). The reaction mixture was purified with a NAP-5 size-exclusion column (5 kDa cutoff, GE Healthcare, Uppsala, Sweden) with a total elution volume of 1 mL PBS. This yielded in 58 MBq of: [^124^I]IgG-scFv_2_ with a radiochemical purity (RCP) > 99%, radiochemical yield (RCY) of 77% and specific activity (As) of 0.14 MBq/µg. RCP was assessed using radio-TLC on iTLC-SG paper (Agilent, SGI0001) with 70% acetone in water as the mobile phase. The retention factors (Rf) were: [^124^I]IgG-scFv_2_: Rf = 0, Free [^124^I]Na: I Rf = 1.

In vitro stability of [^125^I]IgG-scFv_2_ was assessed in PBS at 4 °C and 37 °C, and in mouse plasma at 37 °C. Stability was analyzed by iTLC (mobile phase 70% acetone in water) immediately after purification and after 3 days of incubation, in line with the end-point of the ex vivo and PET studies. In vivo plasma stability of [^125^I]IgG-scFv_2_ was evaluated in App^NL−G−F^ and WT mice injected with the radiolabeled antibody. Plasma samples were collected 3 days p.i. and analyzed by iTLC using the same procedure as described above. RCP was calculated as the percentage of radioactivity retained at the origin, representing intact antibody, relative to total radioactivity on the strip. For in vitro and in vivo stability testing results, please see Supplementary Table S2.

To ensure that the binding affinity of the antibodies had not been affected by the radiolabeling, indirect ELISAs were performed (see Enzyme-linked immunosorbent assays (ELISA) in Materials and methods).

### Ex vivo study of ^125^I-iodinated bispecific antibodies

Mice, under light isoflurane anesthesia, were intravenously (i.v.) injected via the tail vein with 0.4 mg/kg, corresponding to 1.2 ± 0.3 MBq (Supplementary Table S1). Using a syringe needle, the tip of the tail vein was pricked and a blood sample (8 µL) was collected in a capillary tube at the following time points after injection: 30 min, 1, 2, 4, 6, 24 and 48 h. The mice were anesthetized with isoflurane 2, 24, 48–72 h after injection, and a terminal blood sample was taken from the heart, followed by transcardial perfusion with 0.9% NaCl. The terminal blood sample was placed in an Eppendorf tube prepared with heparin. The tube was centrifuged for 5 min at 10,000 × g at 4 °C. Plasma was separated from the blood cells by aspiration. To investigate biodistribution of the radiolabeled antibodies, the brain, lung, liver, kidney, heart, pancreas, spleen, thyroid, skull, muscle, bone and urine, were harvested. The brain was divided into left and right hemispheres, and the left hemisphere was further divided into cerebrum and cerebellum. The brain samples were kept at − 80 °C until further analysis. The radioactivity of all samples was measured with a γ-counter (2480 Wizard™, Wallac Oy PerkinElmer, Turku, Finland). Antibody concentrations were expressed as percent of injected dose per gram tissue (%ID/g). A separate group of six App^NL−G−F^ mice was used to study the binding specificity of IgG-scFv_2_ to TREM2. Half of the mice were administered isotopically unmodified IgG-scFv_2_ at a dose of 20 mg/kg, i.e. a 50-fold higher dose than the tracer dose used for the ex vivo uptake studies described above. Four days later, all six animals were administered an i.v. tracer dose of [^125^I]IgG-scFv_2_ corresponding to 1.8 ± 0.3 MBq (Supplementary Table S1). The brain was isolated at 72 h p.i. as described above. Sagittal brain sections, 20 μm, were prepared for ex vivo autoradiography and exposed to phosphor-imaging plates (Fujifilm) for two weeks. Plates were read with an Amersham Typhoon IP phosphor imager (GE Healthcare), at a resolution of 50 μm, and the images were converted to a false color scale (Royal) in ImageJ (1.54p, Fiji).

### In vivo PET study with ^124^I-iodinated bispecific antibodies

One day prior to the injection of [^124^I]IgG-scFv_2_, the mice to be scanned were given 0.5% NaI in their drinking water to reduce thyroidal uptake of ^124^I. App^NL−G−F^ and WT mice (*n* = 8) were injected with 1 mg/kg corresponding to 6.3 ± 1.0 MBq [^124^I]IgG-scFv_2_ (Supplementary Table S1). After the injection, NaI concentration in the drinking water was decreased to 0.2% NaI. Blood samples (8 µL) were obtained from the tail vein at the following time points after injection: 30 min, 1, 2, 4, 6, 24 and 48 h.

At 72 h after injection, the mice were anesthetized with 3% sevoflurane and scanned in triplicates for 120 min. The scans were performed in a preclinical nanoScan PET/MRI system (Mediso Medical Imaging Systems, Budapest, Hungary) followed by a CT in a preclinical nanoScan SPECT/CT (Mediso Medical Imaging Systems, Budapest, Hungary). After the scans, the mice were perfused, and the radioactivity in the brains and organs was measured by γ-counting according to the procedure described for the ex vivo studies. Images from the Mediso system were reconstructed using a Tera-TomoTM 3D algorithm (Mediso Medical Imaging Systems) with 4 iterations and 6 subsets. All subsequent image processing was performed with Amide version 1.0.6 [46]. PET and CT scans were manually aligned with a T2-weighted mouse brain atlas [47] to quantify activity in regions of interest. Brain regions used for analysis were whole brain, cortex, thalamus, caudate, hippocampus, and cerebellum.

### Brain tissue extraction

The left cerebrum from the injected mice were homogenized with a Precellys Evolution (Bertin Technologies) (4 × 10 s at 5500 rpm) at a 1:5 weight/volume ratio in Tris-buffered saline with 1% Triton-X (TBST) with complete protease inhibitor (Sigma). After centrifugation, 2 h at 16,000 × g, the supernatant was immediately removed and frozen. Pellets from TBST homogenization were dissolved in 70% formic acid (FA) and centrifuged at 16,000 × g for 1 h before collecting the supernatant.

To investigate TREM2 concentrations in brain tissue samples, a sandwich ELISA was performed using half-area 96-well plates (Corning inc.). Plates were coated with primary antibody AF1729 (0.5 µg/mL, R&D Systems) in PBS and incubated overnight at 4 °C. Plates were then blocked with 1% BSA for 3 h at RT, on a shaker. Brain TBST homogenate samples, diluted 1:50 in ELISA incubation buffer, were loaded in duplicates and incubated overnight at 4 °C. Secondary antibody BAF1729 (0.5 µg/mL, R&D Systems) and HRP-conjugated streptavidin (1:3000, Mabtech AB, Nacka, Sweden) were diluted in ELISA incubation buffer. K blue aqueous TMB substrate (Neogen Corp., Lexington, USA) was used to develop ELISA signals and the reaction was stopped with 1 M H_2_SO_4_. The plates were read with a spectrophotometer at 450 nm.

To measure Aβ38, Aβ40, and Aβ42 concentrations in FA-extracted brain homogenate from App^NL−G−F^ mice, the V-PLEX^®^ Aβ peptide panel 1 (6E10) immunoassay (Meso Scale Discovery, K15200E) was used. Samples were neutralized with 2 M Tris and diluted 1:10 000 in assay diluent before being loaded in duplicate onto pre-coated and blocked 96-well plates together with the secondary Aβ antibody 6E10 conjugated to a SULFO-TAG for electro-chemiluminescent detection. After 2 h of incubation, plates were washed with PBS containing 0.05% Tween, and MSD read buffer was added. Plates were read with a MESO QuickPlex SQ instrument (Meso Scale Discovery).

### Statistical analyses

Data are presented mean ± standard deviation (SD). The Shapiro-Wilk test was used to assess the normality of the data distribution. For normally distributed data, Student’s t-test, one-way ANOVA followed by Tukey’s multiple comparisons test or two-way ANOVA followed by Šídák’s multiple comparisons test were used to correct for multiple comparisons. For non-parametric datasets, Mann Whitney test, Kruskal-Wallis test followed by Dunn’s multiple comparisons test was applied. Correlations were analyzed using Pearson correlation tests or Spearman correlation tests (r), and simple linear regression (R^2^). All tests were two-tailed, with a significance level set at 95%. Statistically significant differences were defined as follows: p-value < 0.05 (*), p-value < 0.01 (**), p-value < 0.001 (***), p-value < 0.0001 (****). Graphs and statistical analyses were performed using GraphPad Prism version 10.4.0 (GraphPad Software, San Diego, California, USA) and in BioRender.com.

## Results

### The bispecific antibody formats showed different binding profiles to TfR and TREM2 in vitro

**Fig. 2:**
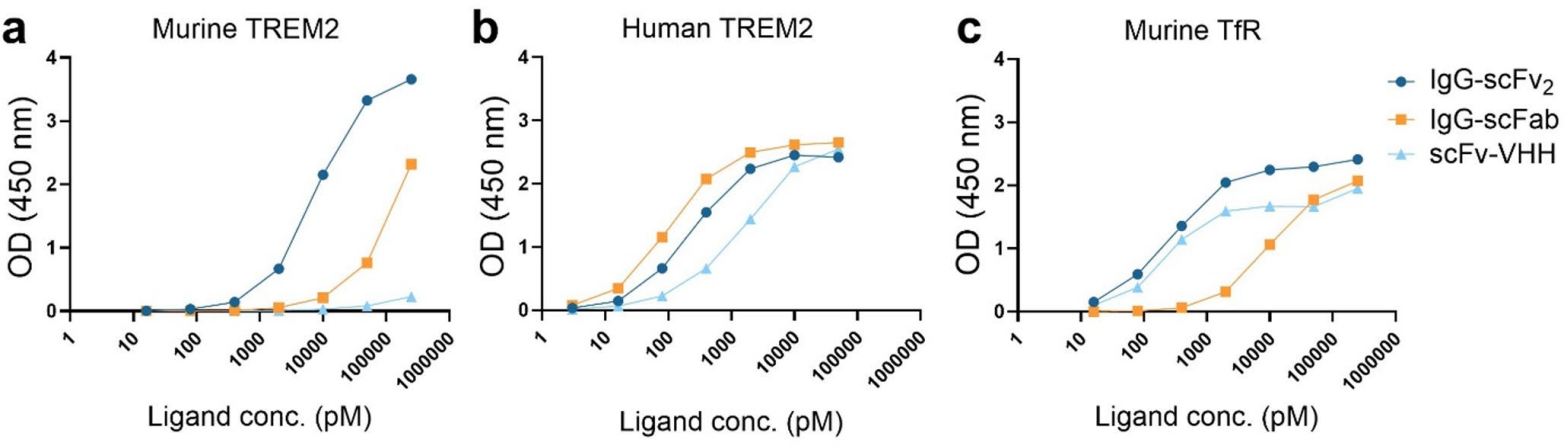
Binding of IgG-scFv₂, IgG-scFab, and scFv-VHH to **(a)** murine TREM2 protein, **(b)** human TREM2 protein and **(c)** murine transferrin receptor (TfR) protein, measured by absorbance in ELISA

Six novel anti-TREM2 antibody formats were produced, with three of the constructs carrying the LALA-PG mutation to attenuate effector functions in vivo (Fig. 1) [43]. The formats lacking the LALA-PG mutation, IgG^wt^ and IgG^wt^-scFv_2_, were not carried forward as their brain uptake was lower than their mutated counterpart (Supplementary Fig. S1a, b). IgG-scFv_2_ showed the strongest binding to murine TREM2 protein, followed by IgG-scFab and scFv-VHH (Fig. 2a). In line with reports of 14D3 preferentially binding human TREM2 protein, all three constructs had comparable in vitro binding to human TREM2 (Fig. 2b) [39]. The three TfR-binders differed somewhat in their affinity to murine TfR protein in direct ELISA (Fig. 2c). IgG-scFv_2_, with bivalent scFv of the TfR antibody 8D3 [27, 41], had the strongest TfR protein affinity, followed by the scFv-VHH, linked to a TfR nanobody [42]. IgG-scFab, carrying a monovalent scFab of 8D3 showed the weakest TfR binding [28].

### Bispecific antibody [^125^I]IgG-scFv₂ demonstrated highest ex vivo brain retention in transgenic mice

**Fig. 3:**
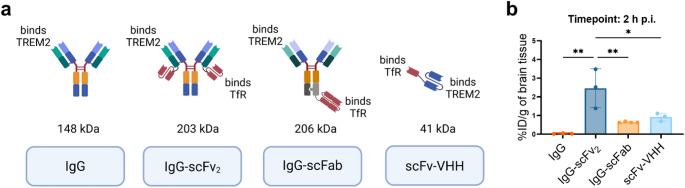
**(a)** Schematic illustration of the anti-TREM2 antibody formats that were carried forward. **(b)** Brain concentration of ^125^I-labeled bispecific anti-TREM2 antibodies, expressed as percentage of injected dose per gram of brain tissue 2 h post injection. WT *n* = 13. One-way ANOVA followed by Tukey’s multiple comparisons test (*p < 0.05, **p < 0.01), mean ± SD

All bispecific anti-TREM2-anti-TfR constructs were able to cross the BBB and enter the brain in WT mice two hours post injection (p.i.) (Fig. 3). In comparison to the control IgG without a TfR-binder, IgG-scFv₂ displayed 67-fold higher brain concentrations, followed by a 25-fold and 17-fold increased brain concentration for scFv-VHH and IgG-scFab, respectively (Fig. 3b).

**Fig. 4:**
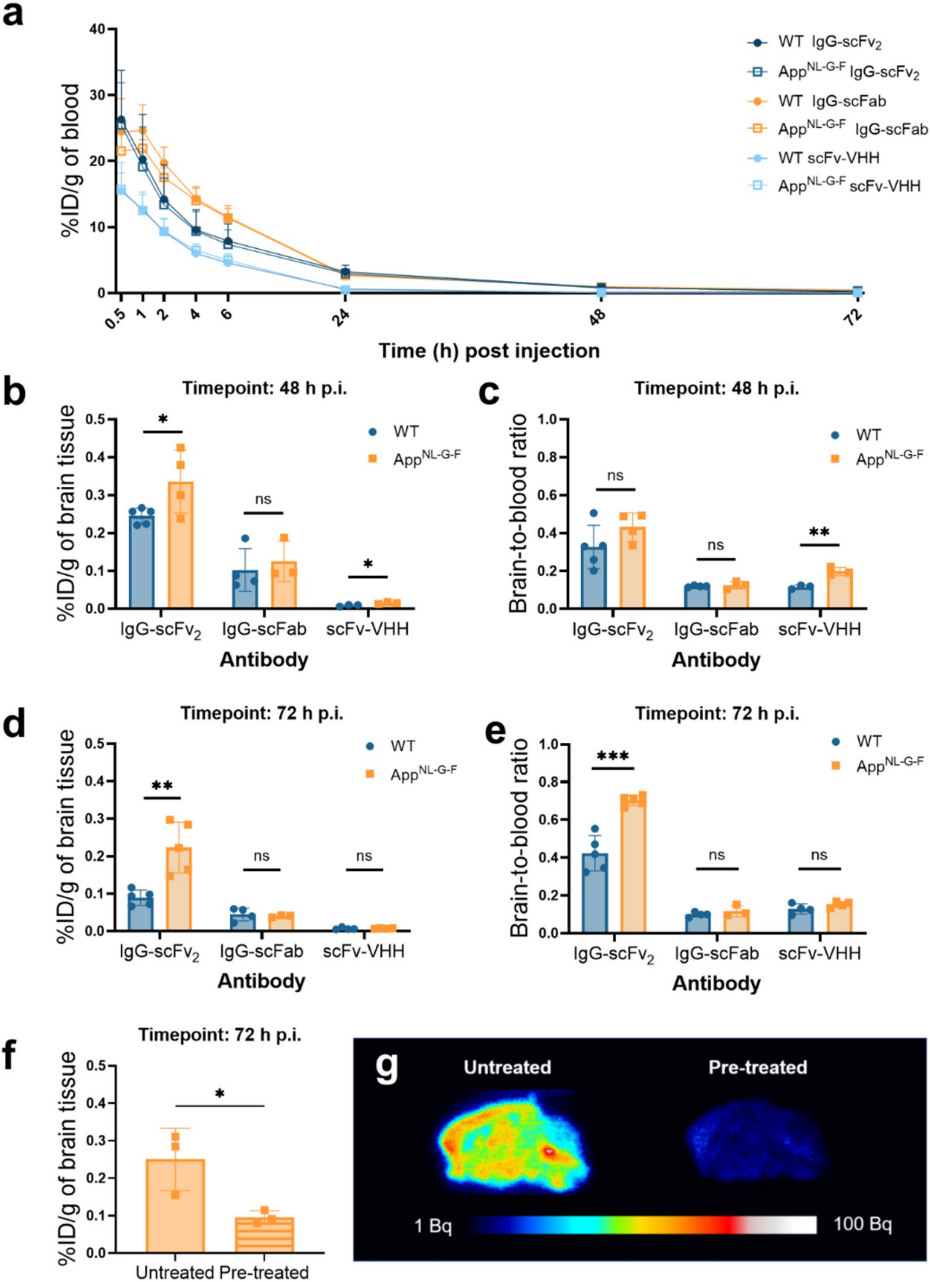
**(a)** Blood-time concentration (%ID/g of blood) curves up to 72 h post injections (p.i.). Brain concentration of ^125^I-labeled bispecific anti-TREM2 antibodies 48 h p.i. **(b)** expressed as percentage of injected dose per gram of brain tissue (%ID/g of brain) and as **(c)** brain-to-blood concentration ratio (App^NL−G−F^*n* = 10, WT *n* = 12). Brain concentration of ^125^I-labeled bispecific anti-TREM2 antibodies 72 h p.i. **(d)** expressed as %ID/g of brain and as **(e)** brain-to-blood concentration ratio (App^NL−G−F^*n* = 12, WT *n* = 13). (**f**) Brain concentration of [^125^I]IgG-scFv_2_ 72 h p.i. in App^NL−G−F^ mice untreated (*n* = 3) or pre-treated (*n* = 3) with a 50-fold higher dose of IgG-scFv_2_ four days prior. (**g**) Representative ex vivo autoradiography showing binding of [^125^I]IgG-scFv_2_ 72 h p.i. in saggital brain sections prepared from untreated and pre-treated litter mates. Student’s t-test (*p < 0.05, **p < 0.01, ***p < 0.001), mean ± SD

Once the ability of the bispecific constructs to cross the BBB had been evaluated in WT mice, App^NL−G−F^ mice were added and the experimental time points were extended to 48 and 72 h p.i. The blood clearance of the bispecific constructs was found to be format-dependent rather than genotype-dependent, with the smallest scFv-VHH exhibiting faster blood clearance p.i. (Fig. 4a). At both 48 h and 72 h p.i., IgG-scFv₂ was able to distinguish App^NL−G−F^ mice from age-matched WT mice (Fig. 4b, d). App^NL−G−F^ mice also had a significantly higher brain-to-blood concentration ratio than the WT mice at 72 h p.i. (Fig. 4e). The brain concentration of IgG-scFab did not significantly differ between genotypic groups at any of the timepoints (Fig. 4b, d). The scFv-VHH displayed higher brain concentrations and a higher brain-to-blood ratio in App^NL−G−F^ mice than in WT mice at 48 h, but not at 24 h (Supplementary Fig. S1c) and 72 h p.i. (Fig. 4b-e). Peripheral biodistribution at 2, 48 and 72 h p.i. is shown in Supplementary Fig. S2.

To investigate the binding specificity of IgG-scFv_2_ to TREM2, App^NL−G−F^ mice were administered a pre-saturating dose of IgG-scFv_2_. The pre-treated mice exhibited significantly lower brain concentrations of [¹²⁵I]IgG-scFv_2_ at 72 h p.i. compared to the an untreated group of App^NL−G−F^ mice, indicating a blocking effect (Fig. 4f). This effect was further confirmed by ex vivo autoradiography, which showed a markedly reduced [¹²⁵I]IgG-scFv_2_ signal in sagittal brain sections from pre-treated mice (Fig. 4g).

### In vivo PET-imaging with IgG-scFv₂ detected elevated TREM2 levels in App^NL-G-F^ 72 h post injection

**Fig. 5:**
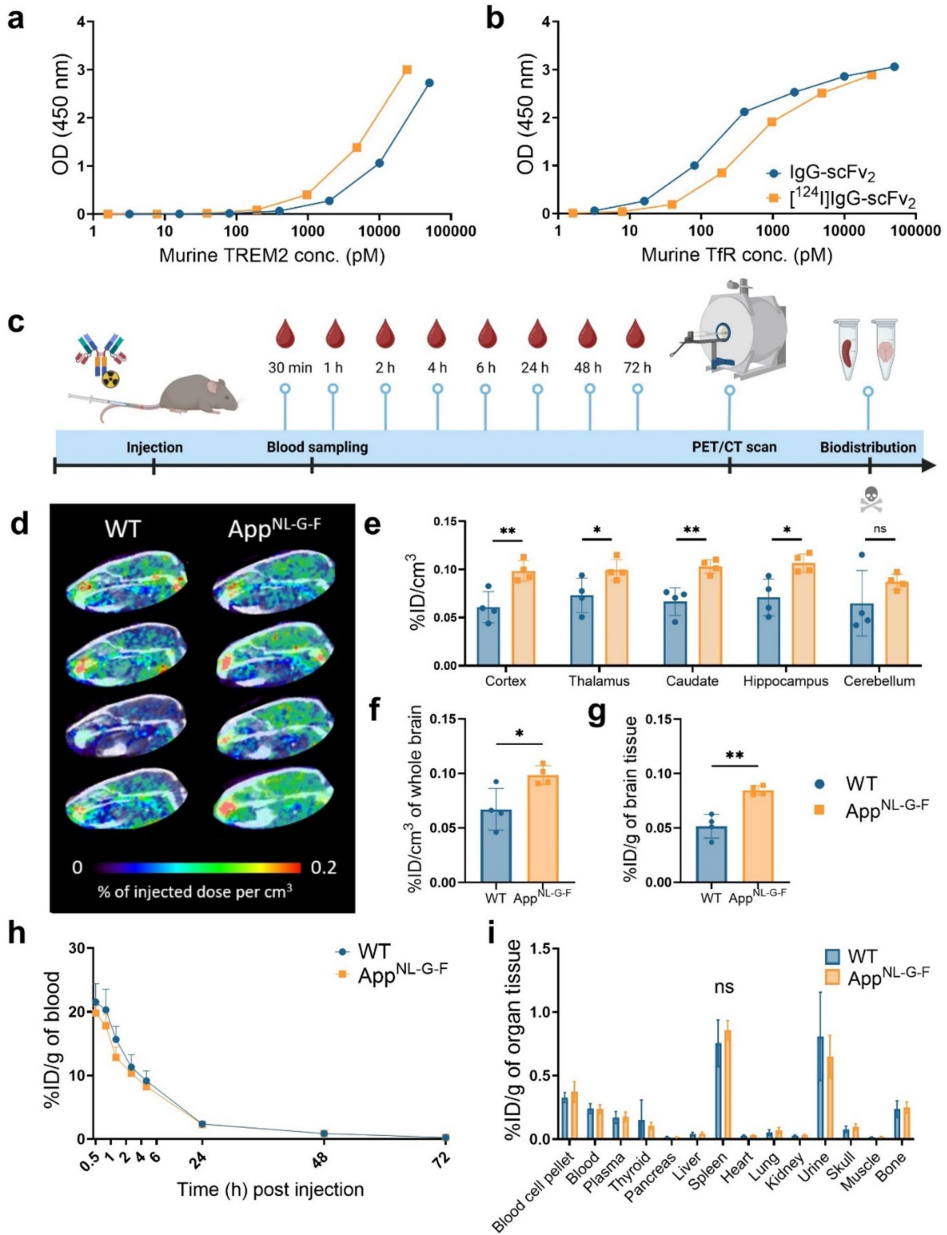
Radiolabeling of IgG-scFv_2_ with ^124^I did not significantly alter the **(a)** binding to murine TREM2 protein or **(b)** murine TfR. **(c)** Experimental workflow for PET-imaging of [^124^I]IgG-scFv_2_ 72 h post injection (p.i.). Blood samples were collected from the tail vein at 30 min, 1 h, 2 h, 4 h, 6 h, 24 h, 48 h p.i. Terminal blood samples were collected from the heart prior to transcardial perfusion 72 h p.i. Radioactivity in the collected organs was measured using a γ-counter. **(d-f**) PET-imaging showed significantly higher in vivo brain concentration of [^124^I]IgG-scFv_2_ in App^NL−G−F^ mice in comparison to WT mice, **(g)** which was confirmed by ex vivo measurements. **(h)** Blood-time concentration (%ID/g of blood) and **(i)** organ biodistribution 72 h p.i. (App^NL−G−F^*n* = 4, WT *n* = 4). Student’s t-test, Two-way ANOVA followed by Šídák’s multiple comparisons test (*p < 0.05, **p < 0.01), mean ± SD

As [^125^I]IgG-scFv_2_ achieved the highest brain concentration and demonstrated the most significant distinction between AD and WT mice in ex vivo studies, this format was selected for in vivo PET imaging of the TREM2 protein. Radiolabeling IgG-scFv_2_ with the PET-compatible radionuclide ^124^I had a relatively minor effect on its binding affinity to either murine TREM2 protein or murine TfR protein (Fig. 5a, b).

App^NL−G−F^ mice and WT mice were scanned 72 h p.i. of [^124^I]IgG-scFv₂ (Fig. 5c). In line with the previous ex vivo results, PET-imaging showed that App^NL−G−F^ mice had significantly higher [^124^I]IgG-scFv₂ brain concentrations than the WT mice measured in the whole brain, cortex, thalamus, caudate and hippocampus (Fig. 5d-f). This was further corroborated by the post mortem ex vivo measured brain concentration (Fig. 5g). App^NL−G−F^ mice and WT mice did not differ in blood clearance or peripheral organ retention of [^124^I]IgG-scFv₂ (Fig. 5h, i).

### Elevated PET signals correspond to elevated TREM2 levels in App^NL-G-F^ mice

**Fig. 6:**
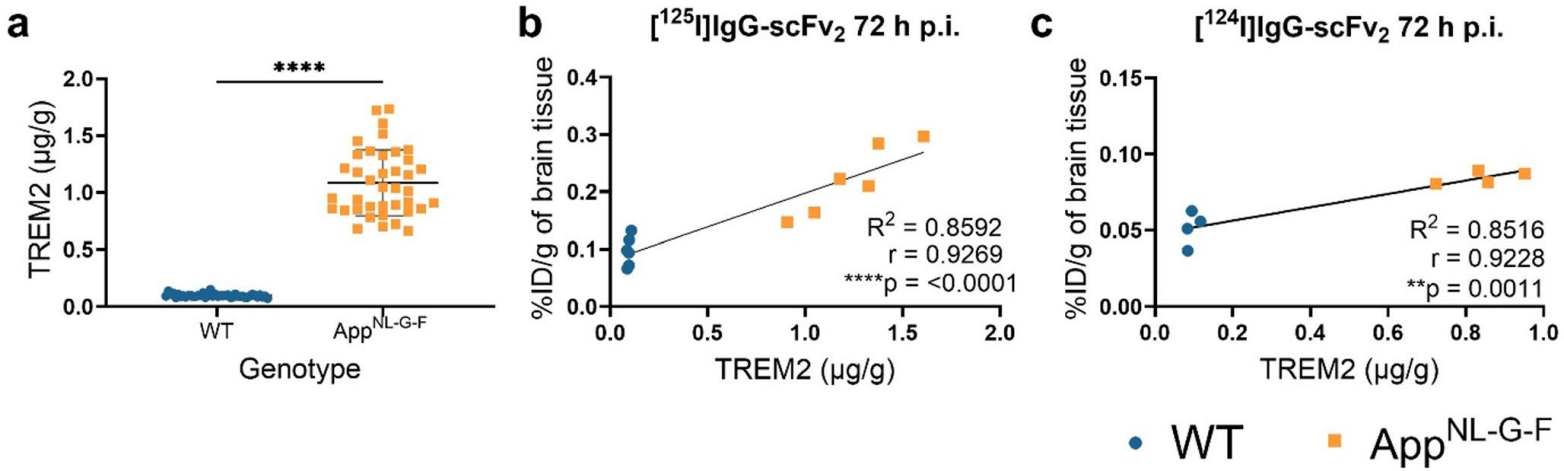
**(a)** TREM2 levels in TBST-extracted brain homogenate were significantly higher in the App^NL−G−F^ mice in comparison to WT mice (App^NL−G−F^*n* = 39, WT *n* = 42). Significant correlations between TREM2 concentrations in brain homogenate and brain concentration (%ID/g) of injected antibody were detected at 72 h p.i. of **(b)** [^125^I]IgG-scFv_2_ (App^NL−G−F^*n* = 6, WT *n* = 6) and **(c)** [^124^I]IgG-scFv_2_ (App^NL−G−F^*n* = 4, WT *n* = 4). Mann Whitney test, Pearson correlation test (r), Simple linear regression (R^2^) (**p < 0.01, ****p < 0.0001), mean ± SD

To confirm that the retention of the bispecific anti-TREM2-anti-TfR constructs and the in vivo visualization of TREM2 using PET-imaging was correlated to increased TREM2 levels, the concentration of TREM2 was measured in the TBST-soluble fraction of brain homogenate prepared from all included animals. App^NL−G−F^ mice displayed considerably higher brain concentrations of TREM2 than WT mice (Fig. 6a). Significant correlations between TREM2 concentrations in brain homogenate and bispecific antibody brain concentrations were found for ^125^I- and ^124^I-labeled IgG-scFv_2_ at 72 h p.i. (Fig. 6b, c).

### TREM2 correlates with Aβ load in App^NL-G-F^ mice

**Fig. 7:**
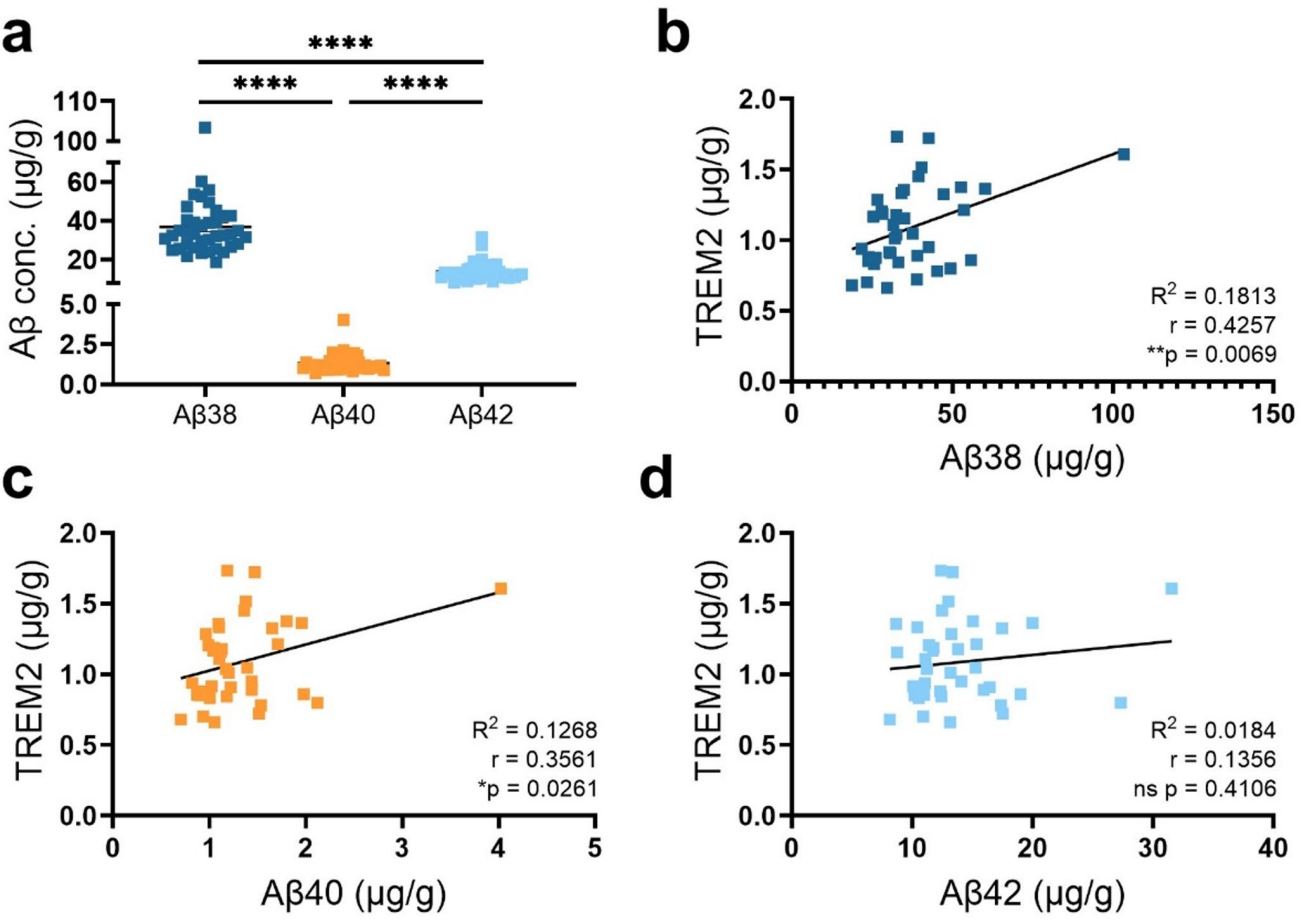
**(a)** Aβ38, Aβ40, and Aβ42 concentrations were measured in formic acid-extracted brain homogenate from App^NL−G−F^ mice (*n* = 39). Significant correlations between TREM2 concentration and concentration of **(b)** Aβ38 and **(c)** Aβ40 were detected in the brain homogenate. **(d)** Levels of TREM2 and Aβ42 did not correlate. Kruskal-Wallis test followed by Dunn’s multiple comparisons test. Pearson correlation test (r), Simple linear regression (R^2^) (*p < 0.05, **p < 0.01, ****p < 0.0001), mean ± SD

In addition to TREM2, Aβ load was measured in the FA-soluble brain homogenate fractions prepared from App^NL−G−F^ mice. Out of the three measured Aβ species, Aβ38 was the predominant isoform, with a 28-fold higher concentration than Aβ40 and 2.7-fold higher concentration than Aβ42 (Fig. 7a). The correlations between TREM2 and Aβ were found to be significant for Aβ38 and Aβ40, but not Aβ42 (Fig. 7b-d). However, these correlations were mainly driven by one mouse with highly elevated Aβ concentrations. When this mouse was excluded from data analysis, only the correlation between TREM2 and Aβ38 remained statistically significant.

## Discussion

Several point mutations in the extracellular domain of TREM2—where the 14D3 antibody used in this study binds—have been linked to neurodegenerative disorders, underscoring the critical role of TREM2 in maintaining brain health and homeostasis [48, 49]. Further, sTREM2 levels in cerebrospinal fluid have been reported to be increased in AD [50]. Therefore, the reported implications of TREM2 in AD pathology have placed the protein at the forefront of efforts to both monitor and treat neuroinflammation using biologics. However, at present, there are no radioligands available for in vivo diagnostic evaluation of brain TREM2 levels, and only suboptimal radioligands available for detection of activated microglia or neuroinflammation in general.

In this study, we produced and compared radiolabeled bispecific anti-TREM2 antibody constructs based on the 14D3 antibody fused with three different TfR binders. Among these, the bivalent IgG-scFv_2_ format exhibited the highest brain retention in transgenic mice with AD pathology, establishing it as the most effective diagnostic radioligand among the formats tested. Notably, at 72 h p.i., the radioligand [^125^I]IgG-scFv_2_ differentiated aged App^NL−G−F^ mice from age-matched WT controls based on ex vivo radioactivity measurements of isolated brain samples. Moreover, when labeled with the PET-compatible radionuclide iodine-124, [^124^I]IgG-scFv_2_ distinguished App^NL−G−F^ mice from WT mice also through PET imaging.

Additionally, higher brain TREM2 levels were detected in App^NL−G−F^ mice compared to WT mice, and a significant correlation was found between elevated TREM2 and the increased PET signal. The App^NL−G−F^ mouse model is characterized by robust and abundant Aβ pathology at a relatively early age [44]. By 14 months, the App^NL−G−F^ mice have substantial brain concentrations of Aβ38, and a high Aβ42 to Aβ40 ratio distinctive of the Beyreuther/Iberian mutation. This pathology is accompanied by neuroinflammation, including microglial activation. TREM2’s expression on the microglial cell surface enables it to act as a sensor—directly interacting with Aβ isoforms. TREM2 has a high affinity for Aβ oligomers, especially soluble Aβ42, and facilitates Aβ internalization [51]. Studies in TREM2-deficient mice have also emphasized the importance of TREM2 in compacting Aβ into a less harmful form [23]. The TREM2-mediated effects in AD pathophysiology, together with its extracellular location, make it a well-suited focal point for microglia-specific PET-imaging.

PET radioligands are ideally based on molecules with fast pharmacokinetics. For example, fast elimination of the radioligand from blood contributes to higher imaging contrast for brain targets. In this study, a smaller scFv-VHH format was therefore evaluated, which indeed displayed faster elimination from blood. Accordingly, the brain concentration and brain-to-blood concentration ratio of [^125^I]scFv-VHH differed between App^NL−G−F^ and WT mice as early as 48 h, i.e. one day earlier than with the larger [^125^I]IgG-scFv₂ construct. However, total brain concentrations were substantially lower with the smaller [^125^I]scFv-VHH compared to the larger [^125^I]IgG-scFv₂. Several factors may explain this: first, the different TfR binder may have contributed to less efficient transport across the BBB. Nevertheless, the concentration of 1% ID/g of brain tissue at 2 h p.i. is relatively good and has proven sufficient for other antibody-based radioligands targeting Aβ [52]. Second, the monovalent form of 14D3 has lower affinity for TREM2, which could decrease retention, especially in mice, as this study and others have shown that 14D3 binds more effectively to human TREM2 than murine TREM2. While the lower affinity of 14D3 for murine TREM2 compared to human TREM2 (76% protein homology) is a limitation of this study, it represents an advantage for future clinical studies, increasing the likelihood of successful translation to clinical applications. Thus, from a translational perspective, the small scFv-VHH should not be ruled out as a potential future diagnostic for humans, but its retention was not enough for detection of elevated TREM2 in mice. The third evaluated construct, IgG-scFab, was based on the Roche Brain Shuttle format [28], comprising a monovalent TfR binder and a full-sized 14D3 IgG. Its initial BBB transport, and consequently, the 2 h concentration, was lower than that of the bivalent TfR binder IgG-scFv₂, yet similar to that of the monovalent scFv-VHH. Interestingly, IgG-scFab showed lower affinity for murine TREM2 than IgG-scFv₂, despite both constructs using the same format for the 14D3 moiety. No such difference was observed in binding to human TREM2. Therefore, although this construct did not distinguish high from low TREM2 levels in mice, it may be effective in detecting elevated TREM2 in humans.

Preclinical PET imaging of TREM2 has previously been explored with a radiolabeled bispecific TREM2-TfR antibody, created by chemically conjugating antibody AF1729, which primarily binds to sTREM2, the soluble form of TREM2, and scFv8D3 [38]. In Meier et al. (2021), tg-ArcSwe mice showed increased retention compared to WT controls based on ex vivo radioactivity measurements in perfused brains; however, this difference was insufficient to distinguish the genotypes by PET imaging. More recently, TREM2 levels were detected using a bispecific antibody in the 5xFAD mouse model expressing human TfR [x viv^39^]. The study utilized a copper-64-labeled 4D9 antibody incorporating a region in the Fc domain that binds to human TfR, following Denali’s Antibody Transport Vehicle (ATV) format. PET imaging with ^64^Cu-labeled ATV:4D9, targeting both soluble and membrane-bound TREM2, gave a pronounced cortical radioligand uptake at 20 h p.i. The study reported higher brain concentrations in 5xFAD mice than in WT at this time point. However, the study also reported high blood concentrations of the radioligand. Furthermore, higher blood concentrations were observed in 5xFAD mice compared to WT mice. At elevated blood concentrations, the radioactivity in the blood volume of the brain can constitute a significant portion of the total PET signal. This could represent a major contributing factor, independent of TREM2 levels, to the increased radioactivity detected in the 5xFAD brain compared to WT when using ^64^Cu-labeled ATV:4D9 PET. Notably, the brain-to-blood ratio of ^64^Cu-labeled ATV:4D9 was observed to be very similar between 5xFAD and WT mice. In contrast, this ratio was significantly elevated in App^NL−G−F^ mice compared to WT when using the radiolabeled 14D3-based IgG-scFv₂ in the present study. Unlike 4D9, the 14D3 antibody specifically targets membrane-bound TREM2. This is likely to enhance the retention of the radioligand in the brain by preventing its clearance through the natural elimination of sTREM2. For a more direct comparison between these two radioligands it would be interesting to use a ^64^Cu-labeled version of the IgG-scFv₂. The above-mentioned study with sTREM2-targeted antibody AF1729 also suggests that binding to sTREM2 is suboptimal, as the retention of the radioligand is likely influenced by the turnover of sTREM2 [38]. Furthermore, Shojaei et al. (2024) included in vitro autoradiography on human brain sections using the 14D3 antibody in the bispecific ATV format, further supporting the clinical translatability of the 14D3-based [^124^I]IgG-scFv₂ findings presented in this present study.

To conclude, we have produced and evaluated three bispecific anti-TREM2 antibody formats that were able to cross the BBB by TfR mediated transcytosis and thus achieve high brain concentrations. The constructs showed binding to both murine and human TREM2 in vitro. Using the bispecific radioimmunoconjugate [^124^I]IgG-scFv₂, PET imaging of TREM2 in the living brain was successful. Utilizing PET imaging to visualize TREM2-associated microglial activation will hold significant diagnostic promise for AD patients and offers a non-invasive method to assess the efficacy of new anti-inflammatory drug candidates.

## Supplementary Information

Below is the link to the electronic supplementary material.Below is the link to the electronic supplementary material.

Supplementary file1 (DOCX 490 KB)

## Data Availability

The datasets used and/or analysed during the current study are available from the corresponding author on reasonable request.
