# Supplementary material for: PET imaging of TREM2 in amyloid-beta induced neuroinflammation
Source: Eur J Nucl Med Mol Imaging. 2025 May 28;52(11):4320–33. doi: 10.1007/s00259-025-07358-0 (PMC12396982; doi:10.1007/s00259-025-07358-0)
Supplement: Supplementary file 1 — Supplementary file1 (DOCX 490 KB) [file 259_2025_7358_MOESM1_ESM.docx]

PET Imaging of TREM2 in amyloid-beta induced neuroinflammation

Amelia D Dahlén^1^, Sahar Roshanbin^1^, Ximena Aguilar^1^, Nadja M Bucher^1^, Sara Lopes van den Broek^1^, Dag Sehlin^1^ and Stina Syvänen^1*^

^1^Department of Public Health and Caring Sciences, Section of Molecular Geriatrics, Uppsala University, Sweden

*Correspondence: stina.syvanen@.uu.se

**Supplementary information**

### Antibody production and purification

#### Cloning

The genes intended for expression were cloned into a pcDNA3.4 vector (Genscript). For IgG-based antibody formats, the light and heavy chains were cloned into separate vectors, while the VHH format was cloned into one vector. GenScript’s recommended Kozak sequence and codon optimization for mammalian expression systems were utilized.

#### Expansion of Expi293 cells

Antibodies were transiently expressed in Expi293F cells (ThermoFisher, A14527), at 37°C, 125 rpm, 5% CO_2_ atmosphere, and 70% humidity in plastic flasks with 0.2 μm ventilated caps (Corning® Erlenmeyer sterile polycarbonate). The cells were maintained in BalanCD HEK293 medium (Fujifilm Irvine Scientific, 91165) supplemented with L-glutamine (4 mM final concentration), in a Minitron™ CO_2_ orbital shaker with 25 mm orbitals (Infors HT, Switzerland).

Cell density was monitored using a Nexcelom Auto T4 cell counter. The cells were split to a density of 0.3 million viable cells (mvc) per mL approximately every four days, upon reaching a density of 3-5 mvc/mL. Cells used for transfection had a viability above 95%, and had not reached a density above 3-5 mvc/mL prior to transfection.

#### Transfection

The day before transfection, the cells were diluted to a density of 0.6 mvc/mL using fresh, pre-warmed BalanCD HEK293 medium supplemented with L-glutamine (4 mM final concentration). The cells were incubated under standard conditions overnight.

On the day of transfection, 0.1% Pluronic® F-68 (VWR, A1288.0100) in BalanCD HEK293 medium was prepared for 20 mvc/mL and pre-warmed to 37°C to reduce shear forces during transfection. Ultrapure Salmon sperm DNA (Invitrogen, 15632011) was added to the pre-warmed 0.1% Pluronic® F-68 media (0.7 µg of DNA per 1 mvc), followed by the addition of plasmids (0.3 µg of DNA per 1 mvc). For IgG antibodies, the heavy chain plasmid and the light chain plasmid were added in a 30:70 ratio.

Cells were pelleted by centrifugation at 220 × g for 8 min at room temperature (RT) using 225 mL centrifuge tubes (Falcon, 352075) in a Centrifuge Eppendorf (5810). In a 125 mL flask, the cells were resuspended at a final concentration of 20 mvc/ml in pre-warmed media containing 0.1% Pluronic® F-68, Ultrapure Salmon sperm DNA and plasmids. Thereafter, L-glutamine (4 mM final concentration) and 3.75 µg of linear 40 kDa PEI Max (Polysciences, 24765–1) was added per 1 mvc and incubated under standard conditions for 3 h.

After incubation, the cells were transferred to a larger flask and diluted to a concentration of 1 mvc/mL with pre-warmed BalanCD HEK293 medium. 0.5 M valproic acid sodium salt (VPA, 2-propyl-pentanoic acid sodium salt, Sigma Aldrich, P4543) was added to a final concentration of 3.5 mM. The transfected cells were incubated on a shaker for six days at 37°C, 125 rpm, in a 5% CO_2_ atmosphere.

#### Harvest of transfected cells

Six days post transfection, cells were counted and harvested by centrifugation at 250 × g for 8 min. For IgG formats, Celpure® P300 (Sigma, 525243) was added to the supernatant at 4 g per 100 mL of media, followed by filtration through a 0.22 μm PES filter bottle top (Thermo Fisher, 595-4520 or 597-4520) and left to reach RT. For the VHH format, Celpure® P300 was added to the supernatant at 1 g per 100 mL of media and filtered through a 0.22 μm PES filter bottle top. Subsequently, 10x buffer W (100 μl/mL of supernatant, Iba Life Sciences, 2-1003-100) and Biolock (18 μl/mL of supernatant, Iba Life Sciences, 2-020-050) were added to the supernatant, and left to reach RT.

#### Purification

IgG antibodies were purified with protein A affinity chromatography using a Hitrap Fibro PrismA column (Cytiva, 17549855) on an ÄKTA GO system (GE Healthcare). The column was equilibrated with phosphate-buffered saline (PBS) pH 7.4, and antibodies were eluted with a linear gradient 0-100% of 0.7% acetic acid pH 2.8. Elution was monitored by measuring absorbance at 280 nm. The knob-in-hole format, IgG-scFab (Fig. 1e), was further purified with size exclusion chromatography (SEC) using a Hiload 26/600 Superdex 200 pg column, and the peak containing monomeric antibody was collected.

The VHH format, that was equipped with a Strep tag, was purified using a Strep-Tactin XT 4 Flow 5 mL (Iba Life Sciences, 2-5024-001). The column was equilibrated with 1x buffer W (pH 8, Iba Life Sciences, 2-1003-100) and 1x BXT buffer (pH 8, Iba Life Sciences, 2-1042-025) was used during an isocratic elution step. Elution was monitored by measuring absorbance at 280 nm, and the column was regenerated using XT-R buffer (Iba Life Sciences, 2-1045-250).

Purified antibodies were neutralized with 1 M Tris (pH 9), and the buffer was exchanged to PBS (pH 7.4) with Zeba spin desalting columns (7kDa, 10 mL, Thermo Fisher, 89894). Protein concentration was measured at A280 using a spectrophotometer (DS-11 spectrophotometer, DeNovix). If necessary, the antibodies were concentrated using Vivaspin 20 centrifugal concentrators (50kDa, PES, VWR, 512-3786).

| **Table S1. Radioligands and injected activity** | | | | |
| --- | --- | --- | --- | --- |
| Radioligand | MBq (SD) per animal | MBq/kg body weight (SD) | MBq/µg of antibody (SD) | Mouse line |
| [^125^I]IgG^wt^ | 1.46  (0.40) | 49.9  (18.0) | 0.11  (0) | App^NL-G-F^ (n = 4, female = 4)  WT (n = 5, female = 2, male = 3) |
| [^125^I]IgG^wt^-scFv_2_ | 1.15  (0.19) | 39.1  (4.7) | 0.10  (0.01) | App^NL-G-F^ (n = 4, female = 4)  WT (n = 5, female = 3, male = 2) |
| [^125^I]IgG | 1.32  (0.22) | 43.5  (3.8) | 0.11  (0.01) | App^NL-G-F^ (n = 5, female = 4, male = 1)  WT (n = 4, female = 3, male = 1) |
| [^125^I]IgG-scFv_2_ | 1.11  (0.42) | 35.2  (14.8) | 0.10  (0.02) | App^NL-G-F^ (n = 13, female = 8, male = 5)  WT (n = 12, female = 7, male = 5) |
| [^125^I]IgG-scFv_2_^*^ | 1.79  (0.32) | 47.6  (1.01) | 0.12  (0) | App^NL-G-F^ (n = 6, female = 4, male = 2) |
| [^125^I]IgG-scFab | 1.40  (0.16) | 41.4  (1.8) | 0.11  (0) | App^NL-G-F^ (n = 6, female = 3, male = 3)  WT (n = 12, female = 6, male = 6) |
| [^125^I]scFv-VHH | 1.07  (0.24) | 34.7  (3.1) | 0.10  (0.01) | App^NL-G-F^ (n = 10, female = 9, male = 1)  WT (n = 13, female = 10, male = 3) |
| [^124^I]IgG-scFv_2_ | 6.29  (0.97) | 164.2  (12.3) | 0.14  (0) | App^NL-G-F^ (n = 4, female = 1, male = 3)  WT (n = 4, female = 2, male = 2) |

***** Blocking study

| **Table S2.** ***In vitro* and *in vivo* stability testing of [^125^I]IgG-scFv_2_** | | |
| --- | --- | --- |
| Condition | % intact tracer  0 h after purification | % intact tracer  72 h after purification |
| *In vitro* | | |
| In PBS, 4°C | 97.2 | 95.6 |
| In PBS, 37°C | 97.2 | 83.4 |
| In plasma, 37°C | 100 | 85.9 |
| *In vivo* | | |
| In plasma | n.a. | 72.7 |

**Supplementary figures**


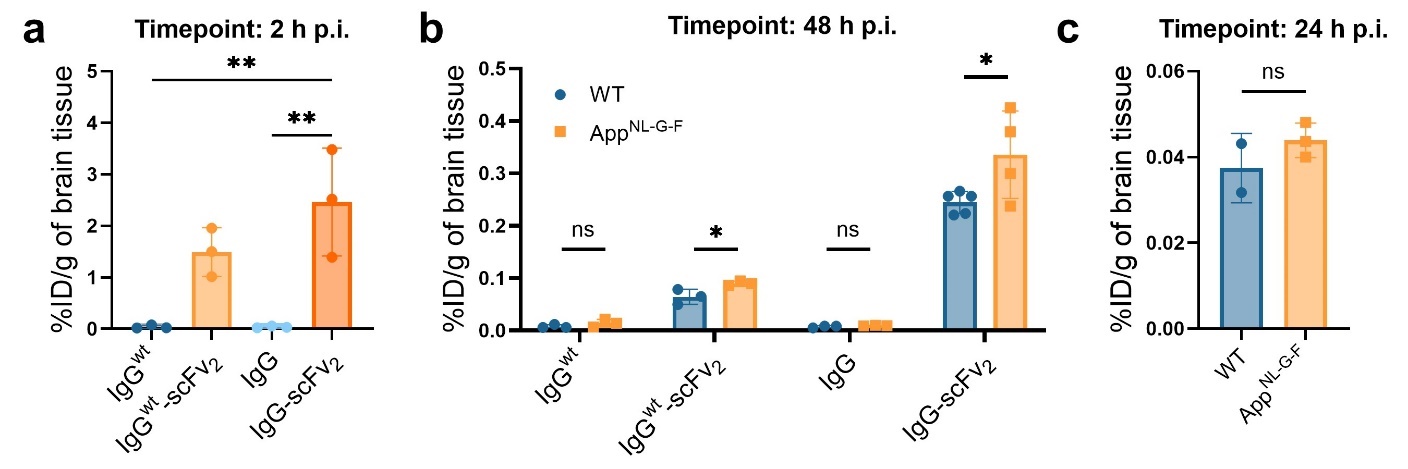


***Fig. S1*** Brain concentration of ^125^I-labeled monospecific and bispecific anti-TREM2 antibodies with and without the LALA-PG mutation, expressed as percentage of injected dose (%ID) per gram of brain tissue at **(a)** 2 h p.i. and **(b)** 48 h p.i. **(c)** Brain concentration of ^125^I-labeled scFv-VHH 24 h p.i. One-way ANOVA followed by Tukey's multiple comparisons test; Mann Whitney test, Student’s t-test, (*p < 0.05, **p < 0.01), mean ± SD


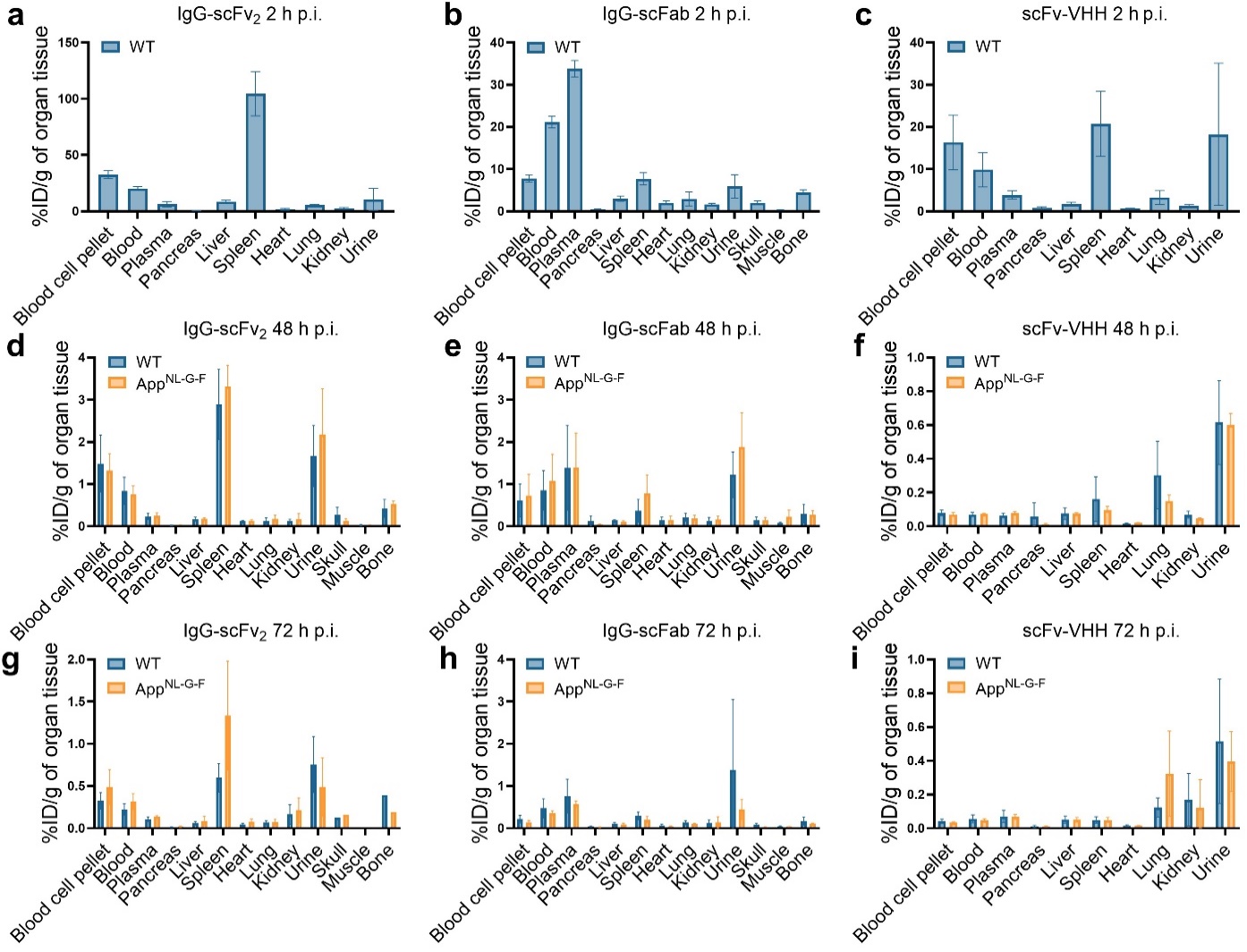


***Fig. S2*** Biodistribution, expressed as percentage of injected dose (%ID) per gram of organ tissue, of ^125^I-labeled IgG-scFv_2_, IgG-scFab and scFv-VHH at **(a-c)** 2 h p.i., **(d-f)** 48 h p.i. and **(g-i)** 72 h p.i., mean ± SD
